# The chronometry of risk processing in the human cortex

**DOI:** 10.3389/fnins.2013.00146

**Published:** 2013-08-20

**Authors:** Mkael Symmonds, Rosalyn J. Moran, Nicholas D. Wright, Peter Bossaerts, Gareth Barnes, Raymond J. Dolan

**Affiliations:** ^1^Wellcome Trust Centre for Neuroimaging, Institute of Neurology, University College LondonLondon, UK; ^2^Nuffield Department of Clinical Neurosciences, Oxford University, John Radcliffe HospitalHeadington, Oxford, UK; ^3^Virginia Tech Carillion Research Institute, Virginia TechRoanoke, VA, USA; ^4^Caltech Laboratory for Experimental Finance, California Institute of TechnologyPasedena, CA, USA; ^5^Swiss Finance Institute, Ecole Polytechnique Federale LausanneLausanne, Switzerland

**Keywords:** decision-making, magnetoencephalography (MEG), risk, neuroeconomics, cortex

## Abstract

The neuroscience of human decision-making has focused on localizing brain activity correlating with decision variables and choice, most commonly using functional MRI (fMRI). Poor temporal resolution means these studies are agnostic in relation to how decisions unfold in time. Consequently, here we address the temporal evolution of neural activity related to encoding of risk using magnetoencephalography (MEG), and show modulations of electromagnetic power in posterior parietal and dorsomedial prefrontal cortex (DMPFC) which scale with both variance and skewness in a lottery, detectable within 500 ms following stimulus presentation. Electromagnetic responses in somatosensory cortex following this risk encoding predict subsequent choices. Furthermore, within anterior insula we observed early and late effects of subject-specific risk preferences, suggestive of a role in both risk assessment and risk anticipation during choice. The observation that cortical activity tracks specific and independent components of risk from early time-points in a decision-making task supports the hypothesis that specialized brain circuitry underpins risk perception.

## Introduction

Risk describes uncertain scenarios wherein chosen actions yield a range of possible outcomes that are quantified by different statistical features in a distribution. On the one hand variance measures outcome spread (uncertainty). On the other hand skewness measures asymmetry, where positive skewness describes distributions with occasional returns well-above average (e.g., casino gambles with high potential winnings) and negative skewness describes distributions with occasional poor outcomes (e.g., rare catastrophic occurrences during routine surgery) (Coombs, 1960; Jullien and Salanie, 2000). Trading off these distinct aspects of risk against potential returns is a central component of value-based choice (Coombs, 1960; Weber and Johnson, 2008), making it likely that evolution has endowed specialized mechanisms for this evaluation.

Considerable evidence indicates involvement of specific cortical and subcortical neural regions in decision-making under risk, ranging from electrophysiological studies in animals (e.g., Fiorillo et al., 2003), lesion-based and neuromodulatory studies (e.g., Knoch et al., 2006; Clark et al., 2008; St. Onge and Floresco, 2010) to neuroimaging investigations in humans with positron emission tomography (PET) (e.g., Ernst et al., 2002), and fMRI (e.g., Critchley et al., 2001; Kuhnen and Knutson, 2005; McCoy and Platt, 2005; Preuschoff et al., 2006; Tobler et al., 2007; Christopoulos et al., 2009; Venkatraman et al., 2009; Mohr et al., 2010b). Recently, several fMRI studies have highlighted scaled responses to different components of statistical risk in particular in posterior parietal and prefrontal cortices (Huettel et al., 2005; Smith et al., 2009; Xue et al., 2009; Symmonds et al., 2010, 2011; Bach and Dolan, 2012). In contrast to previous electroencephalographic or MEG studies of economic decision-making focused on the response to reward feedback (e.g., Gehring and Willoughby, 2002; Hewig et al., 2007), here we investigated the evaluation stage of an economic decision. We capitalize on the temporal fidelity of MEG to study the chronometry of risk responses within these identified parietal and prefrontal regions at a sub-second timescale.

While fMRI allows precise localization of risk-sensitive networks, poor temporal resolution (Kim et al., 1997) limits its ability to inform the temporal sequence of risk appraisal. This is an important lacuna, as risky choices can be evaluated within 1–3 s (Kuhnen and Knutson, 2005; Huettel et al., 2006; Xue et al., 2009), which mandates rapid neural processing of salient statistical features prior to response generation. Moreover, neural responses to risk observed with fMRI might represent an initial encoding of statistical risk or alternatively may represent a risk anticipation signal after option evaluation and any motor preparation signal. To disambiguate these two possibilities we aimed to first test whether it was possible to detect magnetoencephalographic signal changes correlating with risk in the initial evaluation phase of a decision-making task, and secondly to demonstrate whether these signals are detected prior to, or following the formation of signals correlating with an individual's choices. If risk perception is supported by specialized cortical representation, leading to option evaluation and choice, we expect early variance and skewness processing before or concurrent with signal changes predictive of choice. We hypothesized that these signals would be evident in already identified parietal and prefrontal candidate regions. Moreover, an influence of individual risk preference on choice-related signals has also been reported in anterior insula (Christopoulos et al., 2009; Tobler et al., 2009). An early effect of subjective preferences in insula would support an integral role in risk evaluation (Preuschoff et al., 2006), while a late effect would corroborate the alternative theory that insula is involved in an affective anticipation of a risky choice (Kuhnen and Knutson, 2005).

Here we show early variance and skewness cortical responses within parietal and prefrontal candidate regions, as well as preference-dependent activity in anterior insula. Thus, our findings suggest that specialized brain circuitry underpins rapid perception of discrete aspects of risk.

## Materials and methods

### Participants

The study was approved by the Institute of Neurology (University College London) Ethics Committee. Seventeen subjects (mean age: 31; age range: 25–50; 5 male) were recruited for the experiment. One (female) subject was excluded because of metal artifact due to dental work, and 1 (male) subject excluded because of excessive drowsiness and failure to make button-press responses during the experiment.

### Task

To dissociate different components of risk, in terms of dispersion (variance) and asymmetry of outcomes (skewness), we adapted a previously described decision-making task (Symmonds et al., 2011) that controlled for the distribution of possible outcomes. The task, by design, ensured variance and skewness of a set of lotteries could be manipulated independently. Consequently, variance and skewness of gambles were orthogonal factors and this enabled us to test whether neural activity evoked by variance could be segregated in time and place from that evoked by skewness.

**Figure 1:**
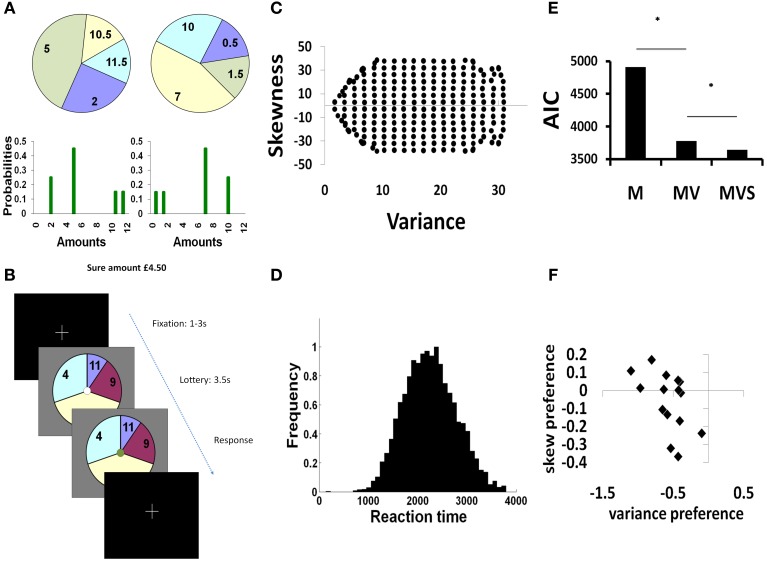
**Experimental paradigm and behavior. (A)** We represented gambles on-screen as pie-charts. The pie chart was divided into four different segments showing possible outcomes from the lottery. The numbers written in each segment show the monetary value of each outcome (in pounds sterling) and the angle subtended by each segment indicated the probability of each outcome occurring. A positively skewed gamble (left) has a small chance of a better than average outcome (the tail of the distribution is to the right). Conversely, a negatively skewed gamble (right) has a small chance of a worse than average outcome (the tail is to the left). Both example gambles have identical variance and expected value. **(B)** Each trial was self paced, with subjects first shown a fixation cross, and then pressing a button to commence the trial. Following initial button press, a pie chart gamble was presented for 3.5 s, during which time the subject was required to make a two alternative forced choice between opting to gamble, or to take a sure amount of money of £4.50. Subjects selected actions by a button press, which was indicated on screen by a color change in the central fixation circle. At the end of the experiment, three trials were randomly selected and played out for real. If subjects had elected to gamble, we resolved the lottery by an on-screen graphic of a red ball spinning around the outside of the pie which stopped at a randomly selected position. **(C)** Plot of the stimulus space used in this study, showing 252 gambles independent in variance and skewness. The non-uniformity at the extremes of variance is limited by the restrictions on stimulus generation (a fixed expected value of 5.95–6.05£, 4 segment pie charts with a minimum probability of 0.1 and probability increments of 0.1). **(D)** Reaction times were normally distributed between ~1–3 s. 99.7% of button press responses occurred after 1 s following stimulus presentation. **(E)** Summed AIC scores for 3 models: mean only (M), mean-variance (MV), mean-variance-skewness (MVS). A lower score indicates a better model fit. The MV model was significantly better than the M model (χ^2^: ^*^p < 1 × 10^−5^), while the MVS model was significantly better than MV (χ^2^: ^*^p < 1 × 10^−5^). **(F)**. Parameter estimates from the MVS model reveal a range of preferences for variance (negative coefficient reflects variance aversion), and skewness (coefficient reflects preference for positive vs. negative skewness).

Participants were required to choose between taking a “sure” (fixed) amount of money or elect to “gamble” (choosing to play a lottery with four potential outcomes). Gambles were represented as pie-charts, where variance and skewness of outcomes varied over a range, while the expected value of gambles was kept constant (Figures 1A,B). On each trial, we recorded choices and simultaneous neural responses (i.e. MEG signal changes) as a function of these changing variables, which allowed us to then determine both when, and where, within a priori regions of interest risk signals are represented.

### Independent manipulation of variance and skewness

We constructed a stimulus set of 252 lotteries (each presented once) where variance and skewness were independent and varied over a range. For every level of variance (16 levels), we independently varied skewness (16 levels, 8 positively skewed, 8 negatively skewed). The expected value of the lotteries was kept constant (between 5.95 and £6.05), and the sure amount alternative remained constant throughout at £4.50. Stimuli were constrained to have 4 outcomes (segments of the pie chart), with outcome probabilities varying in minimum 0.1 increments between 0 and 1 so as to mitigate against probability distortion effects at small probabilities. These restrictions allow the generation of a space of possible lotteries varying in skewness and variance (Figure 1C). Using lotteries with multiple outcomes is crucial in enabling such a dissociation of risk dimensions and consequently we used 4 outcome lotteries throughout to control for perceptual and cognitive variability in task processing.

### Payment

At the end of the experiment, three trials were randomly selected and these were then played out for real. If subjects had elected to gamble, we resolved the lottery by an on-screen graphic of a ball spinning around the outside of the pie until it stopped at a randomly selected position. This procedure was also shown in the practice, to demonstrate the idea that the size of each segment of the pie chart represented the chance of that outcome occurring. Monetary earnings ranged between 13 and £35 (mean £21.17).

### Behavioral modeling

To demonstrate that participants had a behavioral sensitivity to our manipulated risk dimensions of variance and skewness, we compared behavioral models of risk evaluation.

For a given lottery with 4 potential outcomes (*m*_1_, *m*_2_, *m*_3_, *m*_4_), with probabilities *p* = *p*_1_, *p*_2_, *p*_3_, *p*_4_, we define the statistical moments [expected value (EV), variance (Var), skewness (Skw)] of the outcome distribution as:

(1) $$EV=\sum_{n\;=\;1}^{4}m_{n}p_{n}$$

(2) $$Var=\sum_{n\;=\;1}^{4}{\left(m_{n}-EV\right)}^{2}p_{n}$$

(3) $$Skw=\sum_{n\;=\;1}^{4}{\left(m_{n}-EV\right)}^{3}p_{n}$$

In this study, we used the raw statistical moments to define variance and skewness. This use derives from the fact that any utility function can be approximated by preferences for summary statistics using a polynomial expansion (Scott and Horvath, 1980; Müller and Machina, 1987; D'Acremont and Bossaerts, 2008). In practice, the raw and normalized moments are correlated, and our focus here was to draw a distinction between responses to the spread (variance) and asymmetry (skewness) of outcomes, rather than systematically test neural signatures of alternative measures of these statistics.

We analyzed choice data by fitting a linear mean-variance-skewness model (MVS) where individuals are allowed to express different preferences for variance and skewness. To demonstrate sensitivity to both variables of interest, we compared the behavioral fit of this model to two alternatives; a model based on mean difference (M) alone (where subjects only take account of the difference between the sure amount and the expected value of the gamble in selecting actions), and a mean-variance model (MV).

We define the subjective value, or utility (U) of each lottery for our models:

Mean model (M)

(4) U=EV

Mean-variance model (MV)

(5) U=EV+ρVar

Mean-variance-skewness model (MVS)

(6) U=EV+ρVar+λSkw

ρ and λ are free parameters, ρ reflecting preference for variance, and λ reflecting preference for positive vs. negative skewness, respectively.

Our models compare the utility of the lottery with the value of the sure amount (*S*) to generate a trial-by-trial probability of choosing the lottery over the sure amount, using a logistic/softmax function which allows for noise in action selection (by free parameter β).

(7) $$P_{choose\;gamble}=\frac{1}{1+exp(-(1/\beta )(U-S))}$$

We estimated best-fitting model parameters using maximum likelihood analysis, with optimization implemented with a non-linear Nelder-Mead simplex search algorithm in Matlab (Matlab, Natwick, USA). We compared models using the Akaike Information Criterion (AIC), a comparison which penalizes model complexity (Akaike, 1974).

### MEG—experimental setup, recording parameters and preprocessing

We recorded MEG continuously (sample rate: 1200 Hz), using a 274-channel whole-head system (CTF Omega), with participants in a seated position. Stimuli were presented and responses recorded using Cogent presentation software (Wellcome Trust Centre for Neuroimaging, London) written in MATLAB (version 6.5.1, MathWork, Natick, MA). Synchronization of MEG data with the stimulus train was ensured by writing simultaneous timing triggers on each trial to an MEG data channel using the Cogent software (outportb command). Visual cues were projected onto a screen directly in front of the participant. Choices were indicated by pressing a button box with the right index finger. Imaging data were analyzed using Statistical Parametic Mapping software (routines in the academic freeware package SPM8; Wellcome Trust Centre for Neuroimaging, UK, www.fil.ion.ucl.ac.uk/spm).

MEG data were epoched to obtain 1000 ms data segments corresponding to the first second after presentation of the stimulus. This cutoff was chosen as the time window during which stimuli were being evaluated and before button press responses were emitted. On 99.7% of trials motor responses occurred only after this point (Figure 1D). Data were downsampled to 200 Hz, bandpass filtered from 1 to 80 Hz, and baseline corrected. One hundred milliseconds of MEG data prior to presentation of stimulus (when fixation cross was on screen) was sampled as a baseline period.

We performed artifact rejection using an algorithm that rejected all trials where the root mean square (RMS) power was a factor of 10 greater than the average RMS power per trial across subjects.

### MEG—source level analysis

We reduced the dimensionality of our analysis by focusing on four predefined time windows (0–250 ms, 250–500 ms, 500–750 ms, 750–1000 ms) and four frequency bands theta (4–8 Hz), alpha (8–16 Hz), beta (16–32 Hz) and gamma (32–64 Hz), using the multiple sparse prior routine within SPM8 (Friston et al., 2008), with group constraints (Litvak and Friston, 2008). This inverse solution performs an iterative Bayesian optimization to estimate current density on a cortical surface template mesh of several hundred patches, where the mesh is a tessellated template based on the canonical Montreal Neurological Institute (MNI) brain (Mazziotta, 2001), with a single shell template head model. We selected a 250 ms temporal window length given we examine oscillatory responses from 4 to 48 Hz (i.e., minimum one cycle length). Hence we obtained 16 (4 time × 4 frequency) source images per subject per trial.

Our contrasts of interest pertained to our 3 parametric variables of variance, skewness and subject's choice (a categorical variable indicating gamble or sure choice) on each trial. We implemented a standard hierarchical analysis, first estimating within-subjects effect sizes for each of these 3 parametric variables in a general linear model. For each subject, source-localized data were entered into a multiple linear regression against predictor variables corresponding to level of variance, skewness and choice on each trial. All variables were normalized to the range 0–1 and mean-centered. This regression analysis was performed at each of the above time and frequency bins, generating within-subject statistical maps of the regression coefficients corresponding to variance, skewness and choice variables. To make group (between-subject) inferences we then entered each of these 16 (4 time × 4 frequency) source level statistical images per subject into a two-factor repeated measures ANOVA. This second-level ANOVA also included indicator regressors to account for subject effects. Separate ANOVAs were constructed to analyse effects of variance, skewness and choice regressors, respectively.

We were agnostic about the direction of any correlation between decision variables and MEG responses and had no a priori assumptions about the frequency bands in which specific effects would be expressed. For inference, *F*-tests were performed to isolate the main effect over time, collapsing across frequency bands. In other words, we ask where source-localized power correlates with variance, skewness or choice at any of our 4 time windows. Rather than a broadband analysis, we enter trial-by-trial source localized power corresponding to each of our 4 pre-specified frequency bands (4–8, 8–16, 16–32, 32–64 Hz). We analyzed source level induced responses in these windows over subjects using a second-level ANOVA. We used an omnibus test as we had no a priori assumptions about the frequency bands at which effects could be expressed and to avoid multiple comparisons. Note the *F* test denominator degrees of freedom is derived from the dimensions of the second level design matrix, with 15 (subject) × 4 (time bands) × 4 (frequency bands) rows. Subsequent *post-hoc t*-tests were then used to delineate the effect size within each time-bin only in areas expressing a significant main effect.

### Statistical reporting and figures

We restricted our tests to likely cortical regions expressing responses to statistical risk during decision-making, guided by a priori knowledge regarding localization of a risk evaluation network. A meta-analysis of functional imaging studies has identified consistent decision risk (i.e., risk processing before or during choice) responses across several studies in dorsomedial prefrontal cortex (DMPFC), anterior insula and parietal cortices (Mohr et al., 2010a), regions also identified in our similarly designed study where variance and skewness were independently manipulated (Symmonds et al., 2011). We therefore draw inferences in source space by mapping observed profiles of responses in a mask of 6 regions—bilateral posterior parietal, dorsomedial prefrontal, and anterior insular cortices. These were defined using anatomical labels in WFU PickAtlas v2.5 (Maldjian et al., 2003), a software method for generating region of interest masks based on the Talairach Daemon database, converted into MNI spatial coordinates (Lancaster et al., 1997, 2000). We report voxel-wise significant results at a *p* < 0.005 threshold. Brain image figures show second-level SPM-F maps, superimposed upon a canonical brain image, thresholded at *p* = 0.01. Stereotactic coordinates are reported in MNI space (Mazziotta, 2001). Bar plots show effect sizes across time within regions showing a significant main effect.

## Results

Fifteen subjects chose between gambling on a lottery or selecting a sure amount of money in a set of individually presented lotteries where we independently manipulated variance and skewness. On each trial, we recorded choices and simultaneous neural responses (i.e., MEG signal changes) as a function of these changing variables (Figures 1A,B), which allowed us to then determine both when, and where, within a priori regions of interest risk signals are represented. To demonstrate behavioral sensitivity to both variance and skewness, we measured subjective preferences for both these variables using behavioral economic models. We analyzed activity unfolding from stimulus presentation, allowing us to map the temporal sequence of events underlying the initial stage of risk information processing.

### Behavior

Our stimulus set (Figure 1C) was designed such that participants would evenly distribute their choices between gamble and sure amounts. This approach maximizes power for both behavioral fitting and subsequent analysis of MEG activity corresponding to choice. As planned, our subjects on average distributed their choices between gamble and sure options throughout the course of the experiment (mean percentage of gamble choices = 55%, std. 17%). There were few error (missed) trials (0.4% of all trials). Mean response times (RTs) (from stimulus presentation to button press) were 2.26 ± (SD) 0.53 s (Figure 1D).

In designing the paradigm, we aimed to minimize eye movement, and render this uncorrelated with variables of interest. Thus, subjects were required to fixate on a central fixation cross, and pie chart lottery stimuli of the same size were centrally presented with random orientation on the screen. Each lottery had 4 outcomes with numerical amounts placed in identically spaced radial locations (Figure 1A), to ensure that an equal amount of eye movement per trial was required to take in the statistical information, such that saccades would average out across trials and subjects.

We further checked whether eye movements were correlated with variables of interest, recording electro-oculogram (EOG) data (with 3 EOG electrodes placed above, below and lateral to the left eye), in 10 of our participants. We measured eye movement per trial by the magnitude of signal change (from mean) across the EOG channels. The total amount of eye movement in our 1000 ms post-stimulus window of interest was not significantly correlated with the lottery statistics [variance: *t*_(1, 9)_ = 1.2, *p* = 0.27; skewness: *t*_(1, 9)_ = 1.4, *p* = 0.19] or the subject's choice [*t*_(1, 9)_ = 0.9, *p* = 0.37].

#### Sensitivity to both variance and skewness

Individuals' choices were sensitive to both variance and skewness. To formally test this sensitivity we compared a mean-variance-skewness model (MVS), where individuals are allowed to express preferences for both variance and skewness, to a set of alternative decision models (see Materials and Methods). As predicted, a mean-variance-skewness (MVS) model provided a significantly better fit to the observed behavioral data than the alternatives (summed AIC scores: M: 4909; MV: 3778; MVS: 3642); MVS model posterior probability >0.99 (very strong evidence in favor of MVS) (Figure 1E). Our winning MVS model provided subject-specific preference metrics for variance and skewness (Figure 1F). All subjects were averse to variance (average variance preference: −0.56 ± SD 0.25), and 7/15 preferred negative to positive skewness (average skewness preference: −0.06 ± SD 0.16). Beta (temperature) values for the logistic function were low, indicating that choices were well-partitioned by the linear model (average beta = 0.13; SD 0.06).

### MEG source level analysis

We report induced time-frequency responses where our dependent variable is trial-by-trial oscillatory power induced by specific components of a decision. This is in contrast to previous studies using electrographic or magneto-encephalographic recording of economic decision making (Gehring and Willoughby, 2002; Schutter et al., 2004; Hewig et al., 2007; Hedgcock et al., 2010; Harris et al., 2011; Steffen et al., 2011; Yu et al., 2011), which often focus on evoked responses (event-related potentials—ERPs, or fields—ERFs), usually to feedback about decision outcome. While receipt of a reward or loss can generate synchronized event-related signals occurring rapidly following feedback, we focused on time-frequency responses as we were interested in risk evaluation signals evolving over an extended period of hundreds of milliseconds, with considerable potential variability which may curtail detection in evoked responses given signal changes might cancel out in the average.

#### Early encoding of variance and skewness

Our first goal was to test whether we could detect magnetoencephalographic signal changes corresponding to different aspects of risk, and hence characterize this temporal processing during decision-making. We localized induced power in four pre-specified time windows (0–250, 250–500, 500–750, 750–1000 ms) following stimulus presentation, at each of these 4 frequency windows [4–8 Hz (“theta”), 8–16 Hz (“alpha”), 16–32 Hz (“beta”), 32–48 Hz (“gamma”)]. We report results within masked regions of interests to spatially constrain the analyses. These comprise parietal, prefrontal and insula cortex, based on previous investigations (see Materials and Methods).

**Figure 2:**
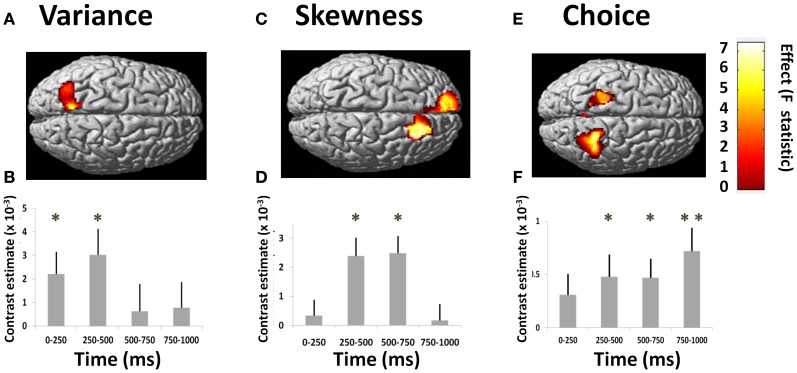
**Responses to risk and choice**. Results of group analysis GLM (two-factor repeated measures ANOVA) of signal power correlating with variance, skewness, and subject's choice. Figures show source-reconstructed induced power parametrically correlating with each of these three regressors. GLM-estimated coefficients for each parametric variable give the estimated slope of the best linear fit, where the null hypothesis at each source is that activity is insensitive to variance, skewness or choice (i.e., the regression coefficient equals zero). Figures show second-level SPM-F image thresholded at *p* < 0.01, superimposed upon a canonical brain (^*^p < 0.01, ^**^p < 0.0001; colorbars show *F*-statistics). *T* = 0 corresponds to time of stimulus presentation. **(A)** Power correlation with variance in left posterior parietal cortex [all effects across 4 frequency bands: “theta” (4–8 Hz), “alpha” (8–16 Hz), “beta” (16–32 Hz), “gamma” (32–48 Hz)]. **(B)** Timecourse of effect, showing significant effects in first 0–250 ms window, peaking at 250–500 ms (variance peak voxel at −12, −56, 52). **(C)** Power correlation with skewness in left and right DMPFC (across all frequencies). **(D)** Timecourses of effects in right DMPFC (skewness peak voxel at 22, 24, 34). **(E)** Power correlation with trial-by-trial choices (gamble vs. sure, across all frequency bands). The effect is seen bilaterally posterior to the central sulcus. **(F)** Timecourse for peak voxel (at 28, −46, 50) shows effects commencing at 250–500 ms, maximal at 750–1000 ms after stimulus presentation.

**Figure 3:**
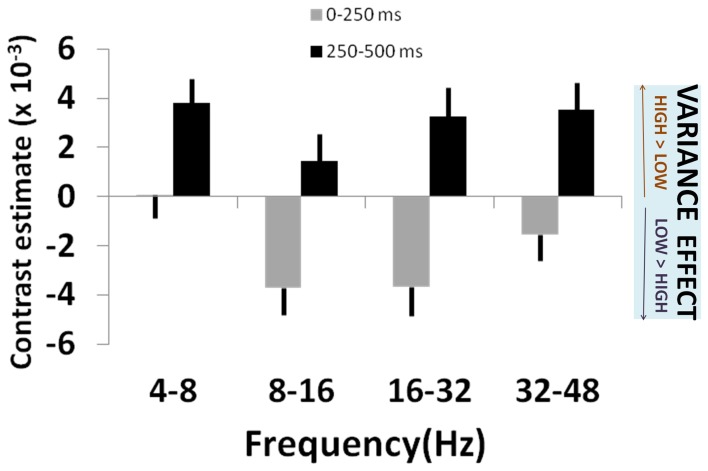
**Variance effects across frequency bands**. Plot of effect sizes for correlation of signal power with lottery variance at 0–250 ms (gray bars) and 250–500 ms (black bars) time windows, for each of 4 specified frequency bands. This corresponds to times at which significant main effects of variance (i.e., across all frequencies) were observed (see Figure 2). Positive contrast estimates reflect greater signal power for high risk (high variance) than low risk (low variance) lotteries. See main text for details and statistical reporting.

We observed an early effect of variance in left posterior parietal cortex [*F*_(3, 210)_ = 4.60, *p* = 0.004; Figure 2A]. This linear modulation of induced power commenced in the initial 0–250 ms following lottery presentation [*t*_(1, 210)_ = 2.32, *p* = 0.011], peaking between 250 and 500 ms [*t*_(1, 210)_ = 2.74, *p* = 0.003] (Figure 2B). On presentation of high (vs. low) variance gambles, power (8–32 Hz) initially decreased between 0 and 250 ms [peak effect: 16–32 Hz, *t*_(1, 210)_ = 2.06, *p* = 0.020], before a broadband increase in power between 250 and 500 ms [peak effect: 4–8 Hz, *t*_(1, 210)_ = 1.75, *p* = 0.040] (Figure 3).

**Figure 4:**
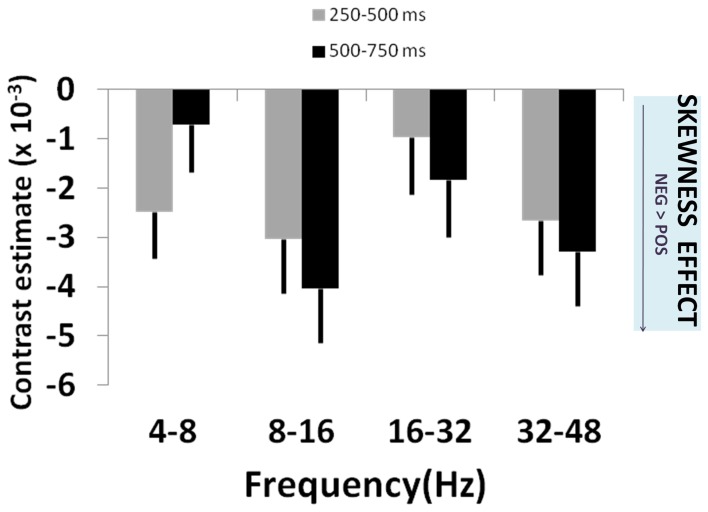
**Skewness effects across frequency bands**. Plot of effect sizes for correlation of signal power with lottery skewness at 250–500 ms (gray bars) and 500–750 ms (black bars) time windows, for each of 4 specified frequency bands. These correspond to the time windows showing significant main effects in Figure 2. The uniformly negative contrast estimates reflect greater signal power for negatively skewed than positively skewed lotteries. See main text for details and statistical reporting.

Gamble skewness significantly correlated with MEG signal power in DMPFC, with a peak effect seen on the right [R DMPFC: *F*_(3, 210)_ = 4.76, *p* = 0.003; Figure 2C], a response present between 250 and 750 ms [*t*_(1, 210)_ = 4.21, *p* < 0.001] (Figure 2D). Within the area of peak effect there was significantly greater power during presentation of negative relative to positively skewed gambles driven by increased alpha (8–16 Hz) and gamma (32–48 Hz) power at 500–750 ms. Thus, the earliest responses to variance are observed 250 ms before the responses to skewness (Figure 4), although there is substantial overlap between the two signals.

These data show that both components of risk (variance and skewness) induce independent scaled modulations of oscillatory activity at similar time epochs in parietal (variance) and prefrontal cortices (skewness). These are regions previously identified as candidate anatomical loci using fMRI, and the MEG data now reveal that these responses occur very rapidly following stimulus presentation.

#### Induced responses to gamble versus sure choices

fMRI studies have consistently reported differential activation during riskier or safer choices (Kuhnen and Knutson, 2005; Christopoulos et al., 2009; Xue et al., 2009; Symmonds et al., 2011), hence we next asked whether choice-specific modulations were evident in the MEG signal. We observed such modulation bilaterally over the central sulcus [right somatosensory cortex: *F*_(3, 210)_ = 7.81; *p* = 0.0001; left: *F*_(3, 210)_ = 5.64, *p* = 0.001; Figure 2E], reaching a peak effect in the 750–1000 ms time window following stimulus presentation [*t*_(1, 210)_ = 3.25, *p* = 0.001; Figure 2F]. This choice-related activity peaked at a later time-point than signals corresponding to decision statistics, suggesting a sequence of cortical processing of statistical risk prior to formation of choice. This choice signal was expressed mainly as a reduction in 8–16 and 32–48 Hz band power prior to choosing to gamble.

**Figure 5:**
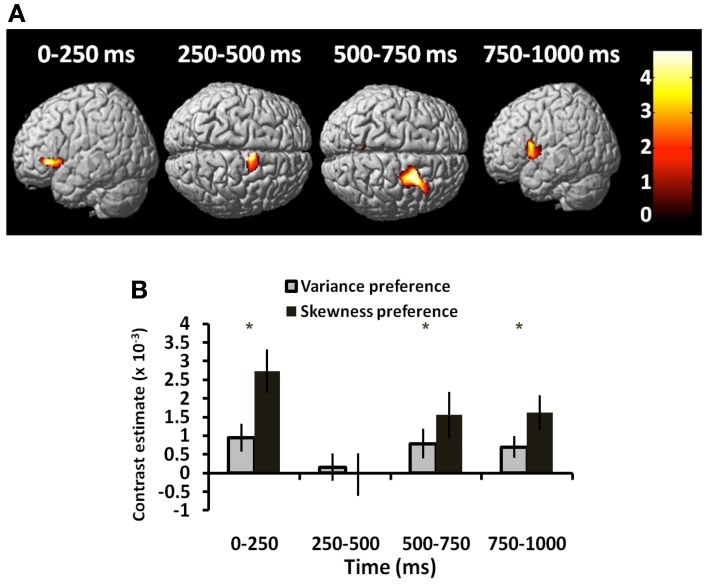
**Choice-dependent responses vary with risk-preference**. Choice-related activity is modulated by risk-preferences. **(A)** Source-reconstructed induced power over 4 time windows showing that choice-related activity (gamble > sure) covaried with both variance and skewness preferences in anterior insula and dorsal premotor cortex (conjunction analysis—colorbar shows *T*-statistic). **(B)** The interaction between neural activity and behavioral preference (both for variance and skewness) plotted for left anterior insula (peak voxel −44, −14, −2). Significant effects (collapsed across frequency bands) were observed in the 0–250 ms window and between 500 and 1000 ms. ^*^p ≤ 0.005.

Exploring this further, we also asked whether choice-related signals were expressed uniformly across subjects, or whether risk-tolerant individuals show a different temporal profile to risk-averse individuals. We found a significant correlation between choice activity and both variance and skewness preference in anterior insula/inferior frontal gyrus (Figure 5A). This interaction of neural activity with behavioral preference was expressed just prior to decision execution in the 500–1000 ms window [conjunction analysis: 500–750 ms, *t*_(1, 54)_ = 2.69, *p* = 0.005; 750–1000 ms, *t*_(1, 54)_ = 2.75, *p* = 0.004], but also in the first time window [0–250 ms: variance coefficient × choice activity: *t*_(1, 54)_ = 2.70, *p* = 0.005; Figure 5B].

**Figure 6:**
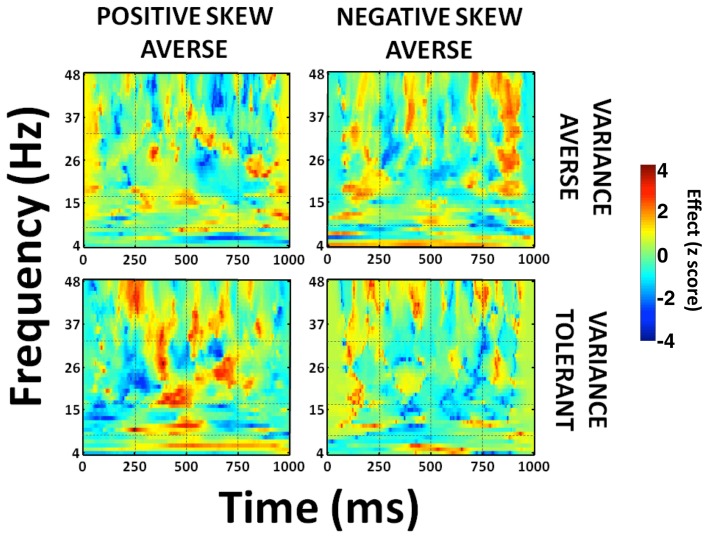
**Time-frequency plot of preference-dependent effects within anterior insula**. Subjects were split into four groups—variance averse/tolerant and positive/negative skew averse individuals (median split). Full time-frequency characterization of the signals in the left anterior insula correlating with choice. This data extraction was performed in a 10 mm sphere centered on peak voxel coordinates (peak voxel MNI coordinates: −44, −14, −2) from our statistical image using a source-extraction routine implemented in SPM. We used a Morlet wavelet time-frequency decomposition with 7 cycles, with a frequency-dependent cycle length as implemented in SPM8 (Kiebel et al., 2005). Gridlines show 4 time windows (0–250 ms, 250–500 ms, 500–750 ms, 750–1000 ms) and 4 frequency bands [“theta” (4–8 Hz), “alpha” (8–16 Hz), “beta” (16–32 Hz), “gamma” (32–48 Hz)]. Late increased beta and gamma activity prior to gamble choices is most evident in variance and negative skew averse individuals, while early theta and beta activity prior to gamble choices is most evident in variance tolerant, positive skew averse individuals. Colorbar shows *z* score values. *T* = 0 corresponds to time of stimulus presentation.

To illustrate these preference-dependent responses, we obtained a full time-frequency characterization using a pseudoinverse to extract regional signals from the anterior insula area displaying maximal effects of our parametric regressors. When we split subjects according to both variance and skewness preference, a general pattern emerged whereby variance tolerant, and positive-skew seeking individuals (tolerant of uncertainty and driven by the potential of large rewards), express an early increase in power prior to a choice to gamble, while individuals with the opposite preference pattern exhibit enhanced activity much later (Figure 6). Thus, insula activity predicts gamble and sure choices, but exhibits a different time course of activity depending upon an individual's specific risk preferences. The early choice-predictive signal in insula occurs concurrently with encoding of variance and skewness in parietal and prefrontal cortices. Conversely, the late effect is consistent with an anticipatory response prior to action execution, with heightened activity prior to gamble choices in individuals who dislike taking risk.

## Discussion

In this study, we map the temporal evolution of induced responses to risk, separating effects of outcome dispersion or uncertainty (variance) and outcome asymmetry (skewness). We show these different aspects of statistical risk are processed as early as 250 ms in separate cortical regions, evident in changes of electromagnetic power in distinct frequency bands.

We suggest that early induced oscillatory activity during lottery evaluation represents encoding of relevant decision variables. These stimulus-locked responses are modulated, per trial, by both variance and skewness consistent with a highly specialized cortical evaluation of risk. By contrast, choice signals seen over the central sulcus (i.e., sensorimotor cortex), occur after these risk encoding effects in parietal and prefrontal cortex, as indeed would be expected from the perspective of a process model of risk perception leading to subsequent evaluation and choice. Notably, these effects are expressed within previously identified risk-sensitive regions (Huettel et al., 2006; Platt and Huettel, 2008; Preuschoff et al., 2008; Mohr et al., 2010b). For example, a linear response to variance in parietal cortex occurs within the first 250 ms, and in DMPFC to skewness, between 250 and 750 ms following stimulus presentation. This pattern supports behavioral evidence (Coombs, 1960; Coombs and Bowen, 1971; Peiro, 1999), and independently corroborates fMRI data (Burke and Tobler, 2011; Symmonds et al., 2011; Wu et al., 2011), that risk is not a unitary phenomenon but is a construct with independent dimensions that are each evaluated within discrete neural networks. PPC has been shown to accumulate perceptual evidence under uncertainty prior to action selection (Huk and Shadlen, 2005; Kiani et al., 2008), and has a general role in numerical and spatial quantification (Hubbard et al., 2005; Piazza et al., 2007) while DMPFC is implicated in encoding probability of loss (Smith et al., 2009; Xue et al., 2010), and is also consistently implicated in risk-processing (Tobler et al., 2007; Bach et al., 2009; Engelmann and Tamir, 2009; St. Onge and Floresco, 2009; Venkatraman et al., 2009; Mohr et al., 2010a). The distributed spatial processing of these risk dimensions corroborates an hypothesis that dispersion and relative hedonic asymmetry of outcomes are supported by separable, specialized, neural processing.

Although responses to risk 250 ms after stimulus onset are clearly rapid, these early responses are similar to processing speeds seen during perceptual decision-making, for example, judging direction in moving dot stereograms (Heekeren et al., 2006), complex discrimination (Fleming et al., 2010), or value comparisons (Milosavljevic et al., 2010; Steffen et al., 2011). Moreover, the parametric changes in induced oscillatory activity we observe are also found to underpin many cognitive processes including sensory perception (Gray and Singer, 1989; Tiitinen et al., 1993; Tallon-Baudry and Bertrand, 1999), comparison (Spitzer et al., 2010), and maintenance in working memory (Romo et al., 1999). It is important to note that individuals are unlikely to be performing an explicit computation of variance, rather a rapid assay of the spread of possible outcomes, for which variance is a surrogate marker (and highly correlated with other measures of dispersion in our stimulus set). Our data make the point that individuals independently respond to both dispersion and asymmetry of outcomes. These responses to variance and skewness overlap in time, although there is a suggestion that signals correlating with variance are detectable prior to those correlating with skewness. While this does not prove temporal precedence, it is interesting to note that this profile mirrors variance being a first-order measure of uncertainty while skewness (mathematically) reflects a second-order attribute (the amount of relative gain vs. loss inherent in a gamble). Although recent investigations in trading behavior under time pressure suggest that skew sensitivity emerges rapidly (Nursimulu and Bossaerts, 2013) it is tempting to speculate that this time delay is sufficiently large to potentially affect outcomes in fast-paced markets (where orders can be transacted within 125 ms).

Our findings where we map out frequency-based responses to risk are exploratory by nature given the dearth of prior research. While this makes it difficult to draw any conclusive inferences about the neurophysiological basis of the expressed effects in specific frequency bands, it is nevertheless notable that this frequency band profile differs for variance and skewness signals. For variance, we note an initial decrease in alpha band power at 0–250 ms. While early decreases in alpha have previously been observed in high-risk social decision-making situations (Qin et al., 2009; Billeke et al., 2012), decreased parietal alpha is also a marker of enhanced attention (Fries et al., 2001) and as all our subjects were variance-averse this relative decrement before selecting lower risk gambles could reflect a top–down attentional effect. This cannot explain the entire picture, and the cause of the subsequent broadband increase in power correlating with variance is less clear. Drawing a tentative parallel with similar cognitive processes, we note gamma band power increases in parietal lobe are associated with mathematical computation (Micheloyannis et al., 2005) but have not previously been shown to scale with specific stimulus attributes as we report here. This rebound increase in gamma signal power with high-risk gambles between 250 and 500 ms echoes findings in perceptual decision-making tasks requiring spatial judgments, where gamma-band enhancements are more evident for easier than more difficult decisions (Kaiser et al., 2007). Similarly, increased beta power has been shown in situations requiring a response inhibition (i.e., here, rejecting the gamble) (Cohen et al., 2008). Increased theta-band power is also seen at this timepoint, an effect that might be related to coupling between encoding and working memory in cross-cortical networks (Sauseng et al., 2005) in decision-making processes. Despite this, it is important to bear in mind that there is no one-to-one mapping between frequency bands and cognitive processes, and our speculations here are almost certainly over-simplistic.

The profile we observe for skewness is markedly different, with a unimodal change predominantly in alpha and gamma power. Increased gamma and alpha power are typically seen during evaluation of task-relevant variables and in maintenance of information in working memory (Tallon-Baudry and Bertrand, 1999; Jensen et al., 2007). Notably, there is greater right frontal alpha power for negatively than positively skewed gambles (i.e., situations with a small chance of a very poor outcome). Increased right frontal alpha power has been suggested to relate to avoidance or withdrawal and negative affect (Harmon-Jones et al., 2010). Skewness captures an implicit relative comparison of better than average to worse than average outcomes, hence we speculate that these alpha power changes may be involved in quantifying an asymmetry in valence of outcomes. Approach-avoidance processes are implicated in decision-making for gains and losses, and are distinct from processing of variance (Wright et al., 2012). However, it remains to be determined if skewness is encoded entirely independently of valence.

The choice signal was expressed mainly as a reduction in 8–16 and 32–48 Hz band power prior to choosing to gamble. Reductions in 8–25 Hz band power (alpha/mu and beta band desynchronization—ERD), as well as a slow D.C. potential shift or magnetic field change (the “readiness potential”) are typically observed prior to voluntary movement (Deecke et al., 1982; Cheyne, 2013). There is also evidence that this desynchronization during motor planning varies dependent upon the movement to be executed (Park et al., 2013), as well as cognitive variables such as response uncertainty (Tzagarakis et al., 2010). This lends further plausibility to our observations of differential signal patterns prior to gamble and sure responses, and the suggestion that these signals reflect specific motor preparation following lottery evaluation.

There is a tension in the theoretical literature between different models of decision-making under risk. Utility theory proposes risk sensitivity as an implicit by-product of a monotonically decreasing utility function (Pratt, 1964; Müller and Machina, 1987). In this schema, a separate processing pathway for risk is superfluous, as risk-preference is purely a consequence of translating objective quantities into subjective values. An alternative model, which has an affinity with financial theory and strongly supported by our data, suggests decisions involve an evaluation of the sufficient statistics or moments of a distribution of outcomes (expected value, variance and skewness). This is a useful heuristic in natural stochastic environments, where it is difficult to encode each possible outcome or state of the world with fidelity. The tracking of summary statistics is also efficient for learning, as it is computationally much easier to update these estimates rather than each outcome and its associated probability separately (D'Acremont and Bossaerts, 2008). While our paradigm is designed to be sensitive to risk effects rather than rule out a utility based encoding of value, our observation of early cortical responses to risk suggest that variance and skewness are relevant perceptual variables in the brain.

The insula has been implicated in risk-processing itself (Preuschoff et al., 2008), the integration of objective risk with subjective risk-preferences (Paulus et al., 2003; Christopoulos et al., 2009; Engelmann and Tamir, 2009; Xue et al., 2010), and in anticipating forthcoming risk (Kuhnen and Knutson, 2005). Intriguingly, we find the temporal profile of insula responses show two distinct effects, an early influence of risk-preference on choice-activity at 0–250 ms after stimulus presentation, and a later effect prior to making a decision at 750–1000 ms. The early effect occurs concurrently with risk encoding in parietal and prefrontal cortices, indicating a possible role in a risk-processing network. The later insula response is consistent with an affective component in risky choice, particularly as it follows rather than precedes choice-sensitive premotor activity. We explored this further, partitioning anterior insula effects in individuals with different risk preference profiles to show positive skew seeking, variance tolerant individuals (similar to casino gamblers who accept risk for the chance of high reward) show strong early responses before both gamble and sure choices rather than simply an early effect preceding a safe default option. More conservative individuals, preferring negative skewness and disliking uncertainty (variance), show dominance of late responses but only before choosing to gamble. Our data therefore hints at a dual role for insula, both at the time of risk encoding and also during entrainment of a motor program for action selection.

There are necessarily limitations to our study. MEG is maximally sensitive to cortical effects, hence we do not explore subcortical risk processing (Knutson et al., 2005; Preuschoff et al., 2006). However, this is compensated for by the fact that MEG allows resolution of risk-sensitive processes at sub-second timescales, measuring the temporal precedence of specific processes. This overcomes problems inherent in inferring causality from fMRI data (Friston, 2009). We focused on the chronometry of responses within the first second following stimulus presentation, as we were interested in processes underlying risk quantification and perception rather than responses locked to action execution. Importantly we show that signals correlating with statistical risk occur very early in decision-making, which strongly suggests a primary process of risk perception rather than evaluative responses emerging after the construction of actions.

## Conclusion

Here we characterize the temporal sequence of neural responses during the perception and evaluation of risk. We show initial rapid processing of salient risk dimensions in PPC followed by DMPFC, manifest as parametric variations in electromagnetic responses correlating with these experimentally manipulated variables. We highlight a temporal dissociation in processing variance vs. skewness, and demonstrate that by mapping the sequence of neuronal activity at a sub-second timescale, it is possible to temporally dissect the dynamic processes that characterize risk evaluation. These early responses to independent statistical components indicate that specialized cortical circuitry underpins rapid decision-making under risk.

### Conflict of interest statement

The authors declare that the research was conducted in the absence of any commercial or financial relationships that could be construed as a potential conflict of interest.
